# Development of BOLD Response to Motion in Human Infants

**DOI:** 10.1523/JNEUROSCI.0837-22.2023

**Published:** 2023-05-24

**Authors:** Laura Biagi, Michela Tosetti, Sofia Allegra Crespi, Maria Concetta Morrone

**Affiliations:** ^1^IRCCS Fondazione Stella Maris, Calambrone, Pisa, Italy, 56128; ^2^Department of Psychology, Vita-Salute San Raffaele University, Milan, Italy, 20132; ^3^Department of Translational Research on New Technologies in Medicine and Surgery, University of Pisa, Pisa, Italy, 56126

**Keywords:** BOLD, infant, motion, MT, vision, visual cortex

## Abstract

Behavioral studies suggest that motion perception is rudimentary at birth and matures steadily over the first few years. We demonstrated previously that the major cortical associative areas serving motion processing, like middle temporal complex (MT+), visual cortex area 6 (V6), and PIVC in adults, show selective responses to coherent flow in 8-week-old infants. Here, we study the BOLD response to the same motion stimuli in 5-week-old infants (four females and four males) and compare the maturation between these two ages. The results show that MT+ and PIVC areas show a similar motion response at 5 and 8 weeks, whereas response in the V6 shows a reduced BOLD response to motion at 5 weeks, and cuneus associative areas are not identifiable at this young age. In infants and in adults, primary visual cortex (V1) does not show a selectivity for coherent motion but shows very fast development between 5 and 8 weeks of age in response to the appearance of motion stimuli. Resting-state correlations demonstrate adult-like functional connectivity between the motion-selective associative areas but not between primary cortex and temporo-occipital and posterior-insular cortices. The results are consistent with a differential developmental trajectory of motion area respect to other occipital regions, probably reflecting also a different development trajectory of the central and peripheral visual field.

**SIGNIFICANCE STATEMENT** How the cortical visual areas attain the specialization that we observed in human adults in the first few months of life is unknown. However, this knowledge is crucial to understanding the consequence of perinatal brain damage and its outcome. Here, we show that motion selective areas are already functioning well in 5-week-old infants with greater responses for detecting coherent motion over random motion, suggesting that very little experience is needed to attain motion selectivity.

## Introduction

The infant visual system is far from complete at birth (Braddick and Atkinson, 2011). Some basic visual properties, like orientation discrimination and color or stereo vision quickly develop in the first 8–10 weeks (Braddick et al., 1980; Held et al., 1980; Braddick et al., 1986; Morrone and Burr, 1986; Clavadetscher et al., 1988; Brown and Teller, 1989; Morrone et al., 1990, 1993). The emergence of these abilities is associated with many developmental events like the maturation of the retinal process, especially the quantum catch sensitivity of photoreceptors (Banks and Bennett, 1988; Movshon and Kiorpes, 1988; Candy et al., 1998; Kiorpes et al., 2003), the increased efficiency in transmission to the central structure thanks to increased myelinizations of optic radiation (Fiorentini and Trimarchi, 1992; Morrone et al., 1996; Dubois et al., 2008), and the maturation of the cortical structure, especially of primary visual cortex (V1; Dubois et al., 2014; Lyall et al., 2015; Gilmore et al., 2018). Although we have a good assessment of perceptual visual property maturation, albeit far from conclusive, and of the anatomic postnatal development of the major gyri, convolutions, and bundle by MRI (Natu et al., 2021), we have limited knowledge of how BOLD develops with age and the cortical functional specialization (Gao et al., 2015; Rajasilta et al., 2020). The few available studies have demonstrated that many visual associative cortical areas are parcellated and active along both the ventral and dorsal pathways by 4–5 months (Deen et al., 2017; Emberson et al., 2017; Kosakowski et al., 2022). We have some preliminary information about the BOLD retinotopic representation in infants ∼5 months old that support the existence of a well-developed retinotopic map (Ellis et al., 2021). However, we are missing most of the information at perinatal time and in the first few weeks of life. This is the crucial stage where major organization of cortical processing takes place, especially for properties like motion analysis that have a fast developmental time (Distler et al., 1996; Bourne and Rosa, 2006; Kiorpes and Movshon, 2014; Arcaro and Livingstone, 2017).

In a previous study we recorded reliable BOLD activity in 8-week-old infants (Biagi et al., 2015). By measuring coherent flow motion against random direction motion, we demonstrated that direction selectivity is well established in many cortical regions and that cortical processing of motion is more mature than commonly assumed (Morrone et al., 1999; Mason et al., 2003; Kiorpes and Movshon, 2014; Nakashima et al., 2019), with selective responses in a network of associative areas similar to that highlighted in adults—the middle temporal area (MT+), visual areas 6 and 6a (V6/V6a), and cuneus area along the medially ascending portion of posterior occipital sulcus (POS) and even associative vestibular-visual area in insula (Kleinschmidt et al., 2002; Frank and Greenlee, 2018). Finally, we observed a delayed maturation of V1, compared with that of these visual associative areas, probably because of the greater microstructural complexity of the primary cortex (Natu et al., 2021). The delayed maturation of V1 is consistent with ERP evidence showing a lateral response foci selective to coherent motion in infants at 5 months and not present in adults (Wattam-Bell et al., 2010). This suggests that the maturation of more posterior occipital areas is delayed and protracted over several months with respect to MT+ and other associative areas.

The network of BOLD responses selective to motion at 8 weeks is consistent with data revealed behaviorally (Wattam-Bell, 1992; Braddick et al., 2005; Braddick and Atkinson, 2009; Maurer and Lewis, 2017). However, it is also well established that even 1-month-old (or younger) infants show defensive motor responses such as blinking and avoidant head movements in response to large-field radial expansion patterns (Ball and Tronick, 1971; Náñez and Yonas, 1994), and it is reasonable to assume that selectivity for motion direction emerges in many regions of the cortex much earlier than 8 weeks, despite the late development of behavioral responses (Wattam-Bell, 1996a, b; Armstrong et al., 2011).

Recording BOLD response in awake children is not feasible at birth but possible at 5 weeks of age (Korom et al., 2022). At this age we demonstrate that the selective BOLD response to coherent motion is already strong and reliable, suggesting a fast maturation of the motion pathways in infants.

## Materials and Methods

### Subjects

The study was approved by Ethics Review Board of Fondazione Stella Maris and the Regional Pediatric Committee (Meyer Pediatric Hospital approval of 14/01/2014). Written informed consent was obtained by all parents before the experiment.

Eight (four females and four males) healthy, full-term (mean 39.9 ± 1.3 weeks; minimum, 38.1; maximum, 42), awake infants, mean age 4.8 ± 0.8 weeks (range, 3.8–5.7 weeks), were scanned by a 1.5T MR scanner (GE Healthcare), all producing reliable data.

Three subjects repeated the experiment after 4 weeks; the results relative to the second scan were published previously (Biagi et al., 2015).

To compare directly data acquired at different ages (8 infants at 4.8 weeks vs 12 infants at 7.7 weeks of full-term subjects in Biagi et al., 2015, born on average at mean 39.8 ± 1.5 weeks; minimum, 37.4; maximum, 42), the methods were the same with regard to both the stimuli as well the acquisition and analysis of MRI data of the previous study.

All infants were assessed by an expert pediatric neurologist by means of the Hammersmith Neurologic examination. The anatomic scans of each MRI examination were inspected by an expert child neuroradiologist to reveal possible anomalies, and all infant brains were referred as normal.

### MRI protocol

The MRI protocol included the acquisition of a three-dimensional (3D) FSPGR (fast spoiled gradient echo) T1-weighted anatomic sequence (TR/TE = 12.28/5.14, isotropic voxel = 1 × 1 × 1 mm^3^), and an fMRI session composed of three distinct GRE-EPI (gradient echo-echo planar imaging) series (TR/TE = 3000/50, flip angle (FA) = 90°, FOV = 240 × 240 mm, isotropic voxel = 3 × 3 × 3 mm^3^). Two series provide the acquisition of 88 time points (4 h 24 min duration) during the presentation of the stimulus in equal duration block design with alternation between two conditions (6 complete periods, equivalent to 12 blocks, each lasting 21 s). Each series included four dummy scans at the beginning of the series to allow the stabilization of MR signal. The alternating conditions were coherent flow motion versus blank in one series and coherent flow motion versus random motion condition in the other.

In the third series, 124 time points (6 h 12 min duration) sampled the fluctuation of BOLD signal during spontaneous sleep of the infants (resting-state acquisition, no stimulus presentation); also for this series, the first four time points were treated as dummy scans and discharged from analysis.

The order of the series was selected on the basis of the sleep or wakefulness of the infants. that is, 3D T1-weighted and fMRI resting state during sleep and functional series with stimulus presentation during wakefulness.

The infants were lying on the scanner table, wrapped in a sheet by an expert neonatal nurse, to reassure the infant and reduce his/her movements. A cotton wool padding, inserted in the auditus of the external auditory canal, and sound-attenuating headphones protected infants' ears. Subject-specific strategies were adopted to minimize the stress level of infants, in accordance with the character and routines of each individual child, for example, use of a pacifier for the child to relax and to fall asleep during anatomic and resting-state fMRI (rs-fMRI) sequences. For each session, one operator, in constant communication with staff in the control room, entered the magnet with the child and assumed the sphinx position, with the operator's arms surrounding the infant's body and hands wrapping the infant's head. During the MR session, if the operator noted strong movements or lack of alertness during a specific functional series, that series was repeated.

### Stimuli and experimental set up

The visual motion stimuli comprised 100 dots, half black and half white, 1.3° in diameter, moving at constant speed (5°/s) with a limited lifetime of 10 frames (∼160 ms at 60 Hz). For coherent motion the trajectory of the dots changed gradually from expansion, inward spiral, rotation, outward spiral, contraction, then repeating the cycle again. The full cycle lasted 2 s. The random motion was constructed using the same dot velocities shuffled randomly over the dots, so it had matched local motion characteristics. The dots covered all the visual field except the central 2°. Every time a dot moved away from the displayed screen or under the fixation circle, it was replotted at a random position. Dot density was kept constant in all displays, and collision between dots was not allowed. All these controls on the dot trajectory ensured that the mean luminance of the stimuli was constant for all types of motion. The mean luminance was 20 cd/m^2^ and contrast 0.85 (Morrone et al., 2000).

**Figure 1.**
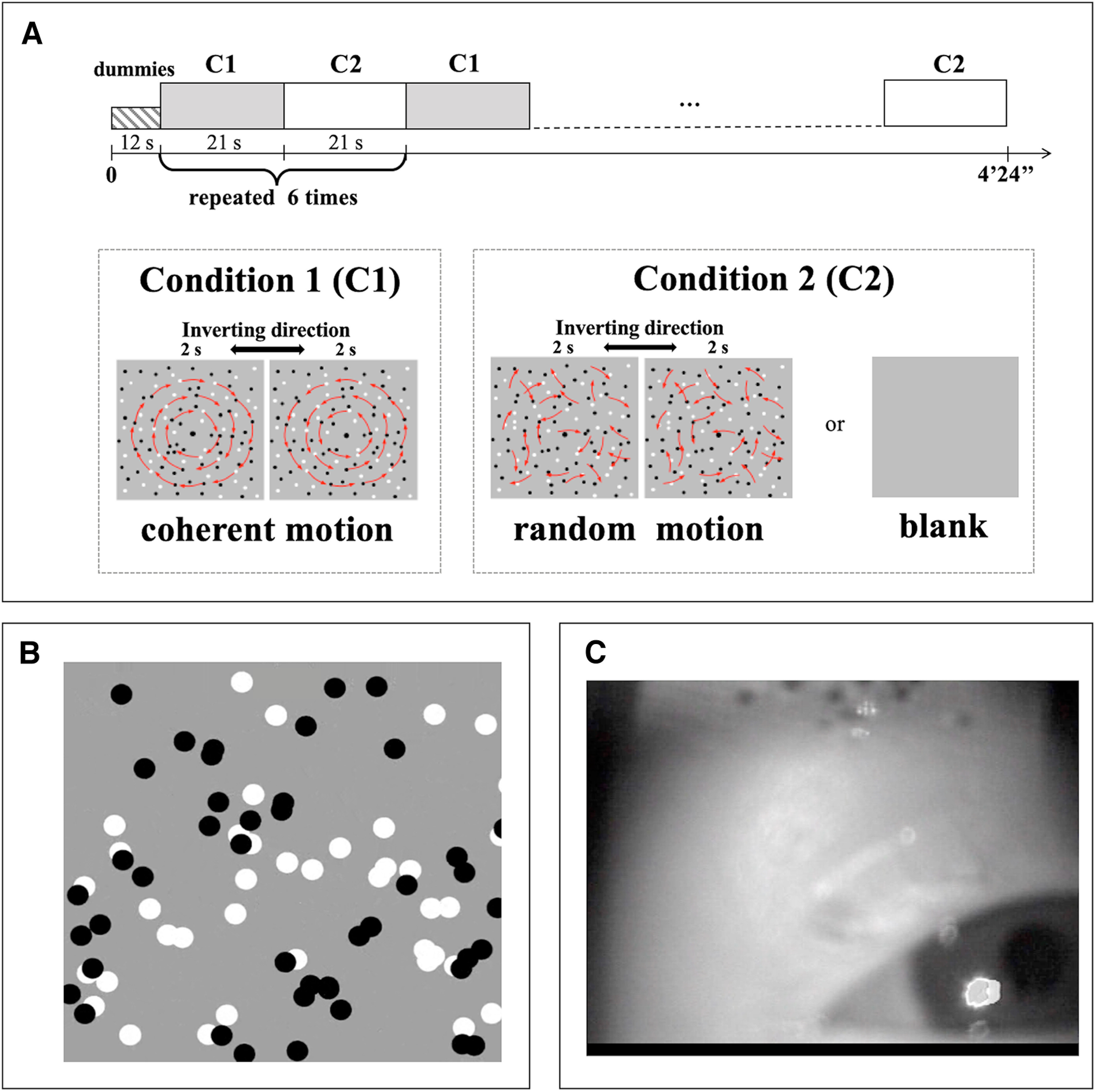
Experimental design. ***A***, Schematic representation of the experimental paradigm. A block design comprising two alternating conditions (C1 and C2) repeated six times each. C1 is always coherent motion; C2 could be either random motion or blank. The coherent motion, starting from clockwise rotation and gradually becoming an inward spiral, does a contraction then an outward spiral, and counterclockwise rotation every 2 s; random noise that has the same local trajectory of the coherent motion (red arrows). ***B***, Example of the display of the visual motion stimuli. ***C***, One frame of the movie recorded by the infrared camera to monitor the eye movements of the infant. The image shows the infant fixing the stimuli and the stimulus reflection on the forehead.

Stimuli were generated in MATLAB (MathWorks) and displayed on LCD goggles (Resonance Technology) positioned inside the head coil ∼10 cm from the infant's eye, giving a visual field of ∼27° × 20°. Fixation of the infant's gaze was monitored by an infrared camera installed within the goggles (sample frequency 60 Hz) and positioned more laterally and oriented slightly more tangentially on the right eye so the pupil could be detected at 10 cm distance. Figure 1 shows a snapshot of stimuli and eye recording. Biagi et al. (2015) has an example of the moving stimuli and of a recording. To assess that the infant's gaze was positioned on the display, we reproduced the position of the goggle and the camera on adults' right eye and evaluated the maximum deflection of the pupil allowing visual stimulation.

During the anatomic and resting state fMRI scans, the goggles were switched off, but the camera remained active to verify the sleep of the infant.

Infant eyes were refracted with retinoscopy at a distance of 87 cm (the virtual image of the goggles is >1 m). All infants were in the normal range between 0 and +2 diopters; given the viewing distance, no additional correction was introduced. The recorded eye movements were analyzed to verify fixation and alertness of the infant during stimuli presentation and sleep during resting-state fMRI series.

### fMRI preprocessing

MRI data analysis was performed with BrainVoyager (BV; Brain Innovation). First, each functional series was inspected for motion spikes or periods of heavy movement. Motion was estimated by calculating the six rigid body parameters (three for translation and three for rotation) for each time point of BOLD signal time course. Time series with head motion >4 (translation) or 5 mm (rotation) were excluded. If periods of movement and stillness were copresent in the same time course, this was segmented into separate intervals. In the following analysis, the segments relative to significant motion were excluded; those relative to stillness were considered as independent shorter functional series. To quantify the extent of the movement, we calculate the framewise displacement (FD), following the methods of Himmelberg et al. (2022) as follows:

$$FD\left(t\right)=\left|\Delta d_{x}\left(t\right)\right|+\left|\Delta d_{y}\left(t\right)\right|+\left|\Delta d_{z}\left(t\right)\right|+\left|\Delta \alpha \left(t\right)\right|+\left|\Delta \beta \left(t\right)\right|+\left|\Delta \gamma \left(t\right)\right|,$$

where $\left|\Delta d_{x,y,z}\left(t\right)\right|$ is defined as the translational shift in millimeters across two consecutive time points along *x, y*, and *z*, respectively, and |Δα(t)|,|Δβ(t)| and |Δγ(t)| are the arc length displacements corresponding to the rotational motion with respect to the three axes, approximating the distance between the center of the head and the cerebral regions of interest (ROIs) to 5 cm (about the distance from occipital pole and head center in infants). Following Himmelberg et al. (2022), we defined a threshold to evaluate movement and counted the number of time points with displacement >0.5 mm.

Table 1 shows the average across subjects of mean head movements along these directions in the used time series, for the three series, (1) coherent flow motion versus blank, (2) coherent flow motion versus random flow motion, and (3) rs-fMRI. The overall movement, as well as FD value, is small in comparison to the fMRI voxel size (3 × 3 × 3 mm^3^), and similar across the two infant and adult groups. Overall, very few time points exceeded the threshold and did not vary across age.

**Table 1.** Estimation of head movement in each group of subjects and for each functional series

		Translation						Rotation						FD		
		*X* (mm)		*Y* (mm)		*Z* (mm)		*X* (degree)		*Y* (degree)		*Z* (degree)				*n* > 0.5 mm
		Mean	SD	Mean	SD	Mean	SD	Mean	SD	Mean	SD	Mean	SD	Mean	SD	Mean ± SD
Blank	Infants 5 weeks	0.16	0.19	0.26	0.30	0.40	0.37	0.79	0.69	0.32	0.20	0.64	0.59	0.21	0.11	4.0 ± 3.3
	Infants 8 weeks	0.24	0.23	0.29	0.26	0.60	0.42	0.82	0.55	0.32	0.24	0.63	0.50	0.28	0.12	5.3 ± 5.8
	Adults	0.18	0.22	0.12	0.11	0.18	0.19	0.29	0.26	0.09	0.10	0.10	0.09	0.07	0.05	0.3 ± 0.7
Random motion	Infants 5 weeks	0.38	0.61	0.19	0.17	0.42	0.36	0.53	0.43	0.43	0.21	0.65	0.25	0.24	0.11	4.4 ± 2.7
	Infants 8 weeks	0.39	0.32	0.35	0.43	0.70	0.64	0.83	0.59	0.76	0.55	0.81	0.79	0.33	0.11	6.1 ± 4.6
	Adults	0.11	0.15	0.07	0.09	0.10	0.07	0.18	0.24	0.09	0.06	0.10	0.08	0.07	0.06	0.5 ± 1.1
rs-fMRI	Infants 5 weeks	0.08	0.07	0.10	0.11	0.28	0.29	0.20	0.13	0.19	0.17	0.22	0.26	0.08	0.03	0.9 ± 0.9
	Infants 8 weeks	0.18	0.22	0.41	0.84	0.48	0.62	0.82	0.93	0.55	1.01	0.62	0.91	0.20	0.12	3.2 ± 3.4
	Adults	0.24	0.24	0.13	0.14	0.18	0.11	0.25	0.22	0.08	0.06	0.09	0.07	0.07	0.04	0.5 ± 0.8

This table reports the estimation of head motion along the six directions (3 translations and 3 rotations) and the measurement of the FD, averaged across all subjects for each functional series (coherent motion vs blank, coherent motion vs random motion, rs-fMRI). The last column reports the number of points in the time series in which FD is greater than the chosen threshold (0.5 mm). Mean indicates the grand mean of values, averaged on all time points of the time series and subjects. SD represents the standard deviation of mean values calculated across subjects.

Given the importance of head movement in resting-state analysis (Power et al., 2012), we report in Table 2 the mean head motion for the rs-fMRI series during sleep for each participant. The head movements during these scans were negligible or absent in all infants, with at most two data points with large movement in the 5-week-old group and nine points in the 8-week-old group.

**Table 2.** Estimation of head movement during the rs-fMRI series for each single subject

		Translation						Rotation						FD		
		*X* (mm)		*Y* (mm)		*Z* (mm)		*X* (degree)		*Y* (degree)		*Z* (deg)				
		Mean	SD	Mean	SD	Mean	SD	Mean	SD	Mean	SD	Mean	SD	Mean	SD	*n* > 0.5 mm
Infants 5 w	5w_1															
	5w_2	0.10	0.04	0.04	0.03	0.15	0.07	0.12	0.07	0.10	0.08	0.21	0.16	0.06	0.03	0
	5w_3	0.04	0.02	0.13	0.04	0.50	0.20	0.18	0.09	0.18	0.06	0.02	0.01	0.08	0.08	1
	5w_4	0.18	0.05	0.32	0.08	0.85	0.23	0.17	0.12	0.57	0.10	0.57	0.14	0.07	0.10	2
	5w_5	0.02	0.01	0.02	0.02	0.11	0.08	0.11	0.06	0.14	0.06	0.02	0.02	0.07	0.06	0
	5w_6	0.15	0.12	0.11	0.06	0.03	0.02	0.49	0.25	0.07	0.04	0.61	0.31	0.13	0.11	1
	5w_7	0.05	0.01	0.05	0.05	0.13	0.11	0.18	0.06	0.11	0.06	0.08	0.03	0.08	0.09	2
	5w_8	0.01	0.01	0.02	0.02	0.18	0.05	0.15	0.09	0.15	0.07	0.02	0.01	0.04	0.04	0
Infants 8 w	8w_1	0.10	0.03	0.16	0.06	0.30	0.16	0.09	0.03	0.24	0.12	0.10	0.04	0.06	0.06	0
	8w_2	0.07	0.03	0.30	0.21	1.49	0.58	1.60	1.00	0.39	0.27	0.34	0.25	0.24	0.22	9
	8w_3	0.08	0.03	0.07	0.04	0.19	0.08	0.54	0.27	0.39	0.12	0.71	0.21	0.18	0.13	1
	8w_4	0.34	0.14	0.18	0.11	0.15	0.12	0.90	0.41	0.25	0.19	0.06	0.21	0.19	0.11	4
	8w_5															
	8w_6	0.10	0.04	0.04	0.03	0.15	0.07	0.12	0.07	0.10	0.07	0.20	0.16	0.06	0.03	0
	8w_7	0.03	0.21	0.01	0.06	0.01	0.07	0.02	0.33	0.03	0.22	0.04	0.42	0.40	0.08	4
	8w_8	0.10	0.08	0.20	0.11	0.40	0.17	1.07	0.66	0.18	0.13	1.22	0.66	0.18	0.13	3
	8w_9	0.71	0.15	2.62	1.06	1.62	0.71	2.84	1.11	3.23	0.66	2.81	1.04	0.38	0.10	8
	8w_10															
	8w_11	0.11	0.07	0.09	0.05	0.05	0.03	0.20	0.12	0.11	0.05	0.09	0.08	0.13	0.08	0
	8w_12															
Adults	A_1	0.09	0.05	0.02	0.01	0.08	0.04	0.03	0.02	0.05	0.03	0.04	0.04	0.03	0.02	0
	A_2	0.54	0.13	0.35	0.12	0.31	0.16	0.69	0.25	0.17	0.20	0.13	0.13	0.11	0.09	1
	A_3	0.07	0.04	0.06	0.02	0.18	0.08	0.11	0.05	0.07	0.02	0.06	0.02	0.03	0.03	0
	A_4	0.67	0.32	0.05	0.06	0.19	0.13	0.17	0.16	0.00	0.07	0.18	0.11	0.11	0.09	1
	A_5	0.15	0.09	0.29	0.18	0.35	0.31	0.36	0.25	0.11	0.05	0.12	0.06	0.11	0.09	0
	A_6	0.00	0.01	0.00	0.05	0.21	0.12	0.05	0.03	0.03	0.03	0.01	0.05	0.10	0.08	0
	A_7	0.19	0.05	0.20	0.07	0.05	0.04	0.41	0.14	0.17	0.04	0.18	0.06	0.06	0.07	2
	A_8	0.18	0.02	0.03	0.02	0.05	0.03	0.15	0.11	0.05	0.03	0.03	0.02	0.04	0.04	0

This table reports the estimation of head motion along the six directions (3 translations and 3 rotations) and the measurement of the FD, of each single subject for rs-fMRI series. The last column reports the number of points in the time series in which FD is greater than the chosen threshold (0.5 mm). Mean indicates the grand mean of values, averaged on all time points of the time series, while SD represents the standard deviation of mean values calculated across all time-points.

**Table 3.** Details of the selected ROIs

	MT+					V6					PIVC					V1-seed					
Infants	*n*	χ	ψ	ζ	Z*	*N*	χ	ψ	ζ	Z*	*n*	χ	ψ	ζ	Z*	*n*	χ	ψ	ζ	Z* rm	Z* b
1	224	−36	−41	7	2.39	70	−18	−63	19	2.34	75	−18	−25	13	1.97	167	−3 ± 2	−56	4	1.7	3.1
	113	30	−42	5	2.88	354	7	−55	15	2.97	80	24	−24	10	1.96						
2	178	−29	−54	1	3.94	213	−11	−60	21	2.61	134	−33	−21	11	2.51	219	−4 ± 2	−59	0	2.37	4.9
	324	28	−61	5	2.43	90	12	−65	17	2.7	101	31	−24	11	2.34						
3	73	−37	−55	−3	3.79	195	−6	−62	23	3.89	61	−22	−23	10	4.35	262	−6 ± 4	−68	−9	1.06	2.5
	64	23	−60	−3	3.72	186	4	−61	17	2.23	70	19	−24	12	2.39						
4	234	−41	−40	2	3.09	146	−8	−56	21	1.96	77	−24	−18	16	1.98	145	−1 ± 3	−67	−10	1.6	2
	279	31	−47	4	3.48	63	7	−57	25	1.96	121	28	−22	13	1.98						
5	87	−31	−51	−2	1.98	238	−10	−59	18	2.1	118	−23	−23	12	2.35	239	3 ± 2	−64	−16	0.12	2.2
	162	36	−51	−3	1.99	130	8	−57	22	1.97											
6	625	−38	−50	4	2.88	209	−12	−61	19	4.17	126	−32	−21	13	2.81	66	−1 ± 3	−54	−4	0.55	2.1
	105	37	−43	1	2.65	229	12	−57	18	2.41	77	22	−25	16	3.29						
7	100	−33	−57	5	2.17	165	−9	−66	16	2.65	68	−29	−28	17	1.98	195	−4 ± 4	−62	−13	2.81	2.2
	308	37	−55	3	2.61	148	6	−64	17	1.97	189	30	−30	18	1.97						
8	68	−42	−44	3	3.29	54	−10	−56	20	1.98					2.07	118	−3 ± 3	−63	−10	0.58	2.7
	96	37	−53	3	2.85						68	23	−19	13							
Mean 5 weeks	199	−36	−49	2		161	−11	−60	20		94	−26	−23	13		176	−2 ± 6	−62	−7		
	181	32	−52	2		171	8	−59	19		101	25	−24	13							
Mean 8 weeks	251	−36	−51	0		204	−11	−63	19		139	−27	−28	10		310	−2 ± 5	−62	−11		
	275	32	−50	1		274	12	−63	17		149	26	−26	11							

For each ROI, this table reports the number of voxels, the position in millimeters in the AC–PC template, and the significance of the BOLD responses. For V1, Z*rm refers to the coherent motion versus random motion (rm), Z*b to coherent motion *versus* blank (b) contrast. For each subject, the first and the second rows correspond to the ROI in the left and right hemisphere, respectively.

For all infants, we were able to select more than half of the periods for each fMRI series (six periods maximum) and more than half length of the complete recorded run for rs-fMRI (120 time points), 5.4 ± 0.9 periods (range, 3.5–6 periods) for coherent versus random flow motion, 4.4 ± 1.0 period (range, 3.5–6 periods) for coherent flow motion versus blank, and 97 ± 22 data points (range, 75–120) for resting state. The remaining periods were discarded because of head motion, sleep during stimulus presentation, or awaking during rs-fMRI. fMRI data preprocessing included mean intensity adjustment to compensate for interscan intensity differences and temporal interpolation and was resampled to compensate for slice-dependent time differences (sinc function), 3-D motion correction (sinc interpolation), and high-pass temporal filtering (GLM-Fourier approach, two cycles). Functional data were coregistered on the three-dimensional anatomic T1-weighted images by using an affine alignment with the standard BV nine parameters (three for translation, three for rotation, three for FOV scale). Infant anatomic datasets were in turn transformed into the anterior commissure–posterior commissure (AC–PC) coordinate system by applying a rigid transformation (six parameters; three for translation and three for rotation).

### Data analysis

For each functional series, a GLM model approach was used to analyze BOLD responses, modeling a regressor of interest and six spurious movement regressors (outputs of the 3-D motion correction procedure). The regressor of interest was calculated by the convolution of the stimulus profile, built as a series of boxcar functions, with a gamma variate function for the hemodynamic response.

The GLM analysis of the BOLD signal registered during the flow motion versus blank stimulus was used to detect activity along the right and left calcarine sulci, labeled as V1-seed (*p* ≤ 0.01). As in Biagi et al. (2015), two correlation maps were calculated at a low conservative threshold (*p* < 0.05), by cross-correlating the BOLD signals of V1-seed ROI with those of all other brain voxels, using 0 and 3 s (one TR) as temporal delays. A mask for further GLM analysis was obtained by the union of these two correlation maps.

The BOLD responses to coherent versus random motion stimulus were analyzed within the combined mask to limit the number of relevant voxels in the GLM analysis. A threshold of *p* ≤ 0.05 was used. The foci (positive or negative) were localized by a neuroradiologist expert in pediatric and neonatal imaging and labeled as in Biagi et al. (2015) as follows: MT+ area (including both MT and Medial Superior Temporal; MST), V6, posterior insular vestibular cortex (PIVC), V1-seed. Table 3 reports the coordinates of the center of mass, the number of activated voxels, and the respective *Z*-score (Z*) for each ROI. The coordinates correspond to the distance (in the three dimensions and in millimeters) of the ROI center of mass from the AC point, once the brain was transformed in the AC–PC coordinate system.

For each stimulus (coherent flow motion vs blank or vs random flow motion) and for each subject, the signal time course was extracted from each ROI.

The percentage of BOLD signal change was obtained for each subject averaging across periods of repetition, and a mean BOLD response was calculated, averaging across subjects. To evaluate the individual mean BOLD, we averaged the extracted signal over four TRs (from the third to the sixth inclusive), corresponding to the period between 9 and 18 s after the beginning of the stimulus.

We also evaluated the signal-to-noise ratio (SNR) and the phase of the BOLD response by performing an FFT on the extracted time course before averaging. SNR was defined as the amplitude of the ratio of the fundamental harmonic and the root-mean-squared amplitude of the two frequencies closest to the fundamental (Boynton et al., 1999). The phase of the BOLD response is a good measure of the hemodynamic delay. The results of FFT analysis is reported in 2D polar plots, where for each point, the distance from the origin indicates the SNR, and the angle (in degrees) represents the phase of the fundamental frequency response. For stimuli with these time characteristics, a standard hemodynamic model corresponds to a phase value of 64°. To compare the phases of the responses between infants of different ages and to calculate statistical significance, for each group, the average and SD were calculated via vector computation by using the resultant of the vector sum and the distances of each vector data point from it.

As previously described in Biagi et al. (2015) the resting-state series were used to study the functional correlation between the ROIs.

In the group of 4.8-week-old infants, only one child did not fall asleep during the MR session, so seven resting-state fMRI datasets are available (in Biagi et al., 2015, we had nine datasets on 8-week-old infants). For each subject, a 7 × 7 symmetric correlation matrix was obtained, calculating for the ij-element the cross-correlation between the BOLD signals registered during resting state in the ROI reported at i-row and in the ROI at j-column (where i, j = 1, …, 7; 1 = MT R, 2 = MT L, 3 = V1, 4 = V6 R, 5 = V6 L, 6 = PIVC R, 7 = PIVC L).

Two mean correlation matrices were obtained averaging the single-subject matrices for infants at 5 weeks and 8 weeks. As no statistical differences were found between the two age groups for any pair of ROIs, a mean correlation matrix was calculated by averaging 13 datasets (counting once the infants who repeated the experiment) to compare with the adult one.

To achieve a value of significance for each value in the mean correlation matrices, for each group of subjects the signals of all single subjects were normalized and concatenated, obtaining the signal of the aggregate subject for each ROI. The calculation of cross-correlation of these signals between each pair of ROIs corresponds to the average across subjects of the correlation matrices and gives the significance of the values in terms of *p* value. Statistical analysis (two-tailed *t* test) was then performed between correlation matrices of the two groups of subjects (infants vs adults).

## Results

To assess the maturation of BOLD motion response, we recorded the BOLD response to the same stimuli used in the previous study but at the younger age, between 4 and 5 weeks old instead of 7- to 9-week-old infants. We also attempted to record younger infants, but the difficulties in keeping the infants alert and awake for the time necessary to complete the scans has proven too difficult and after two failures we decide to increase the limit to 4 weeks.

We used the same procedure for identifying ROIs as the in previous study so that the results between the two age groups could be directly compared. This allowed us to monitor a developmental trajectory of the responding cortices.

We first located the regions along or close to the calcarine sulcus responding significantly more strongly to moving stimuli then to blank stimulus by using a standard GLM with the canonical hemodynamic function. We calculated a mask of all brain cortical regions that are connected with these regions, by computing the correlation between the time course of each voxel with the temporal profile of the responses of these regions to the motion versus blank stimulus. The mask profiles were very similar between the two age groups, although a quantitative test is difficult given the difference in brain size and gyrification (Cusack et al., 2018). We then labeled in the mask the reliable responses to coherent versus random motion. This procedure allowed the elimination of some spurious activity associated with noise and the reduction of the number of multiple comparisons when calculating the correction for statistical threshold. These procedures allowed the location of many of the important area participating in the analysis of flow motion, like MT+, V6, PIVC, precuneus, and others (Morrone et al., 2000; Cardin and Smith, 2010; Biagi et al., 2015; Mikellidou et al., 2018).

**Figure 2.**
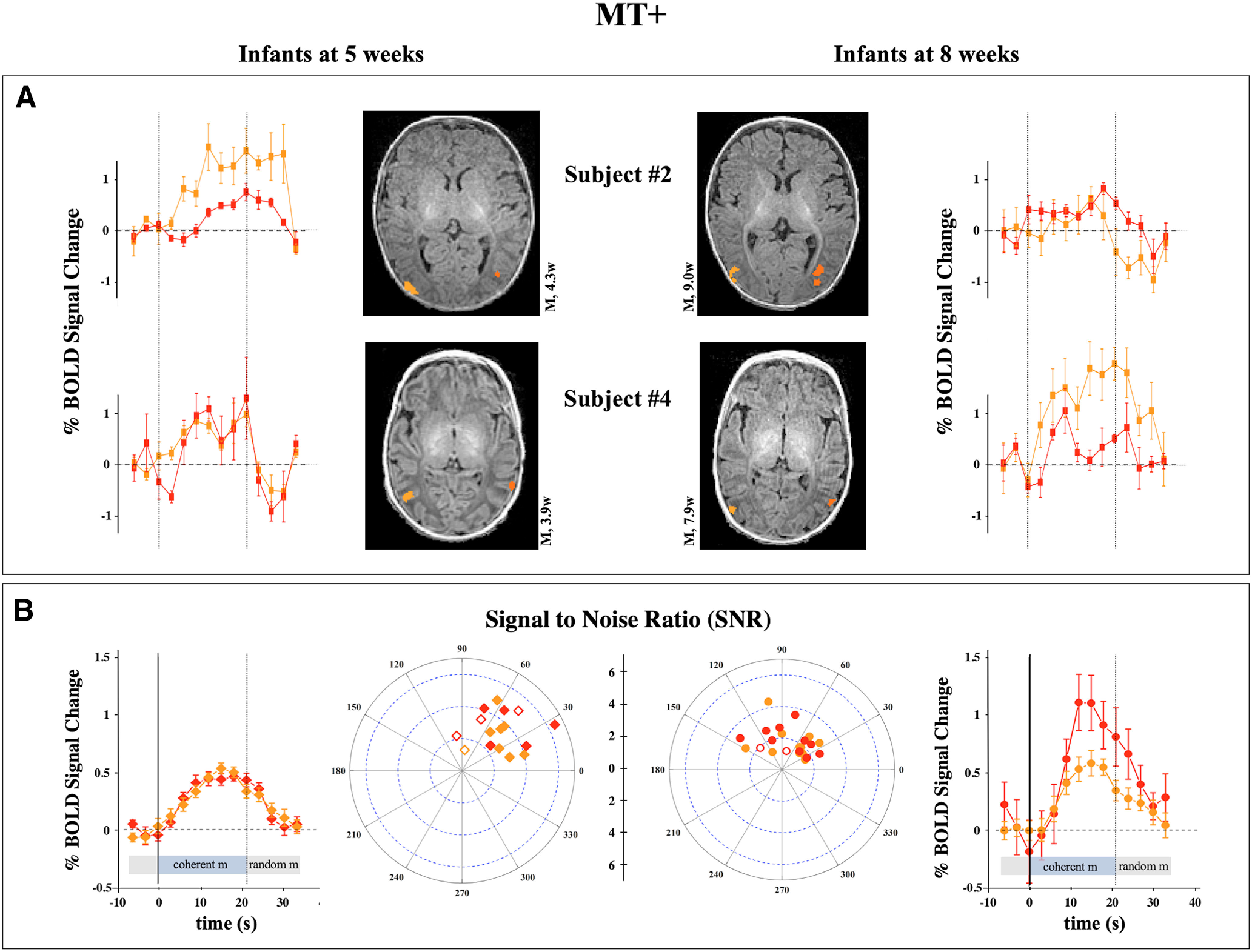
Responses of MT+ to coherent versus random motion. ***A***, Example of two of the three subjects that were recorded twice at about 5 (left) and 8 (right) weeks of age in response to coherent versus random motion. The anatomic localization of the area is similar and consistent with the localization of the MT+ in adults (images are in radiological convention). The curves are the averages of the time-courses in each ROI over good periods, and error bars are the standard deviations over periods. ***B***, The BOLD response averaged across all subjects around 5 (left) and 8 (right) weeks of age. The error bars are the standard deviations across subjects. The bar under the averaged time course marks the period of presentation of coherent flow motion (light blue) and of random flow motion (light gray). The 2D scatter plot shows the SNR and phases of the response estimated from the frequency analysis (see above, Materials and Methods). A significant anticipation of phase and of response amplitude was observed at an older age. Open and solid symbols in the 2D plot indicate responses that are significant at *p* < 0.05 or *p* < 0.01, respectively. The dark and light orange traces show the BOLD modulation of the left and right hemispheres, respectively.

For three infants, we were able to record their response at 5 and 8 weeks of age. In these three infants a clear preference for coherent motion over random motion (putative MT+) was present along the posterior portion of the inferior temporal sulcus. The responses were present both in the left and right hemispheres. About 4 weeks later, we observed similar responses at similar anatomic locations (Fig. 2*A*). The extent (at same *t* threshold), the amplitude, and the hemodynamics of the responses are comparable at the two different ages for these three subjects. Repeating the analysis across all infants, the amplitude of the BOLD response shows a small but significant increase in the older age (8 weeks, 0.72 ± 0.16; 5 weeks, 0.40 ± 0.06; *p* = 0.023). However, neither the extent of the activation nor the signal-to-noise ratio of the BOLD responses were different between the two groups. Despite these similarities, we observed a shorter time to peak of the response corresponding to clustering of the points on the 2D polar plot toward a phase of 46° ± 37° in the 5-week-old group against 74° ± 32° in the older group, consistent with an anticipation of the hemodynamic response during development (Arichi et al., 2012; Fig. 2*B*). Overall, the data show that the selectivity of the BOLD response of MT+ does not change substantially between 5- and 8-week-old infants and that a similar amount of noise is present in the recording of the two infant groups.

**Figure 3.**
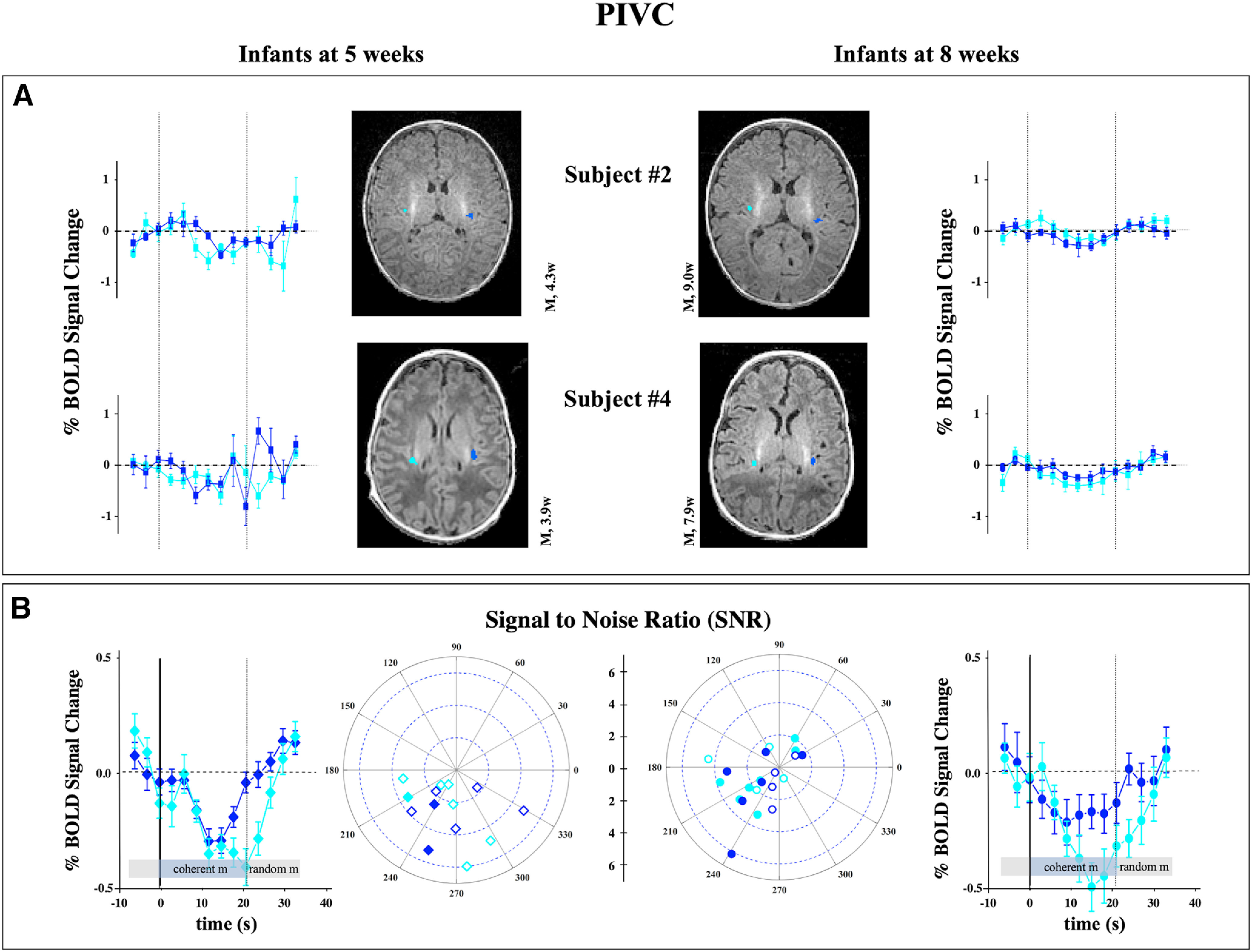
Responses of PIVC to coherent versus random motion. ***A***, ***B***, Same analysis on two longitudinal recordings (***A***) and of the full subject population (***B***) as detailed in Figure 2 for the vestibular associative area PIVC, located in the posterior part of the Sylvian fissure (Frank and Greenlee, 2018). The response for this area is stronger to random motion than to flow motion and similar between the two groups. The average BOLD signal change across all subjects is −0.30 ± 0.13% in the 5 week infants and −0.27 ± 0.08% in the 8 week group (*p* = 0.57).

Another important associative multimodal area that responds well to coherent flow motion is a vestibular cortex in the posterior portion of the insular sulcus, referred to as PIVC. Also, for these areas we observed the same pattern of results, with no observable maturation trajectory between 5 and 8 weeks in the three infants studied longitudinally (Fig. 3*A*). No difference emerged in the extent of the ROI, SNR, and in the amplitude across the two age groups of the entire subject population. The only difference is a small and just significant change of the phase of the responses (8 weeks, 214° ± 46°; 4 weeks, 250° ± 36°; *p* = 0.02) with faster hemodynamic for the older age (Fig. 3*B*).

**Figure 4.**
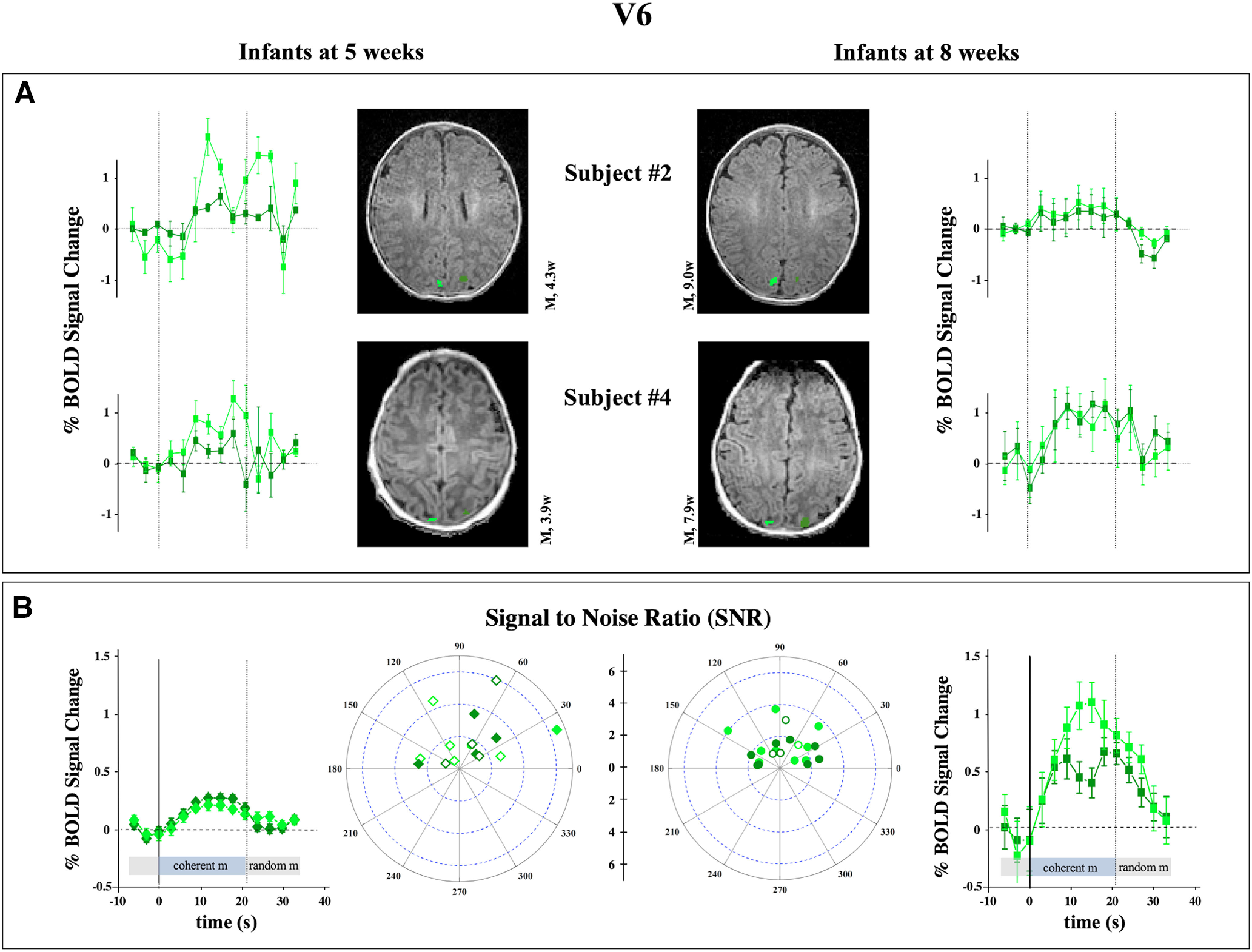
Responses of V6 to coherent versus random motion. ***A***, ***B***, Same analysis of two longitudinal recording (***A***) and of the full subject population (***B***) as detailed in Figure 2 for a visual dorsal associative area located in the parietal-occipital sulcus above the most peripheral representation of dorsal V2 and V3 (Pitzalis et al., 2006). This area is subject to a strong maturation between the two ages with an increase of extent and BOLD modulation of the ROIs.

This pattern of results is somewhat different for cuneus areas. We were able to locate an area that in adults would be labeled as V6. In the longitudinal subjects, the responses are noisier in the younger group (Fig. 4*A*). Similarly, the average BOLD modulation across all subjects is greater in the 8-week-old infants, with a mean response equal to 0.75 ± 0.17% against a mean response of 0.23 ± 0.04% for the 5-week-old group (*p* < 0.001; Fig. 4*B*). Also, the extent of the area is significantly larger (Table 3; *p* = 0.043), suggesting that this region has undergone a maturation during the 4 weeks between recordings. Other more ventral cuneus cortical areas along the medial portion of the posterior-occipital sulcus, which were clearly labeled in the 8-week-old group (Biagi et al., 2015), could not be revealed in the 5-week-old infant, despite the region being included in the correlation mask. For the ventral cuneus area along the posterior-occipital sulcus, no response to coherent versus random motion was observed at this age after relaxing the statistical significance threshold.

**Figure 5.**
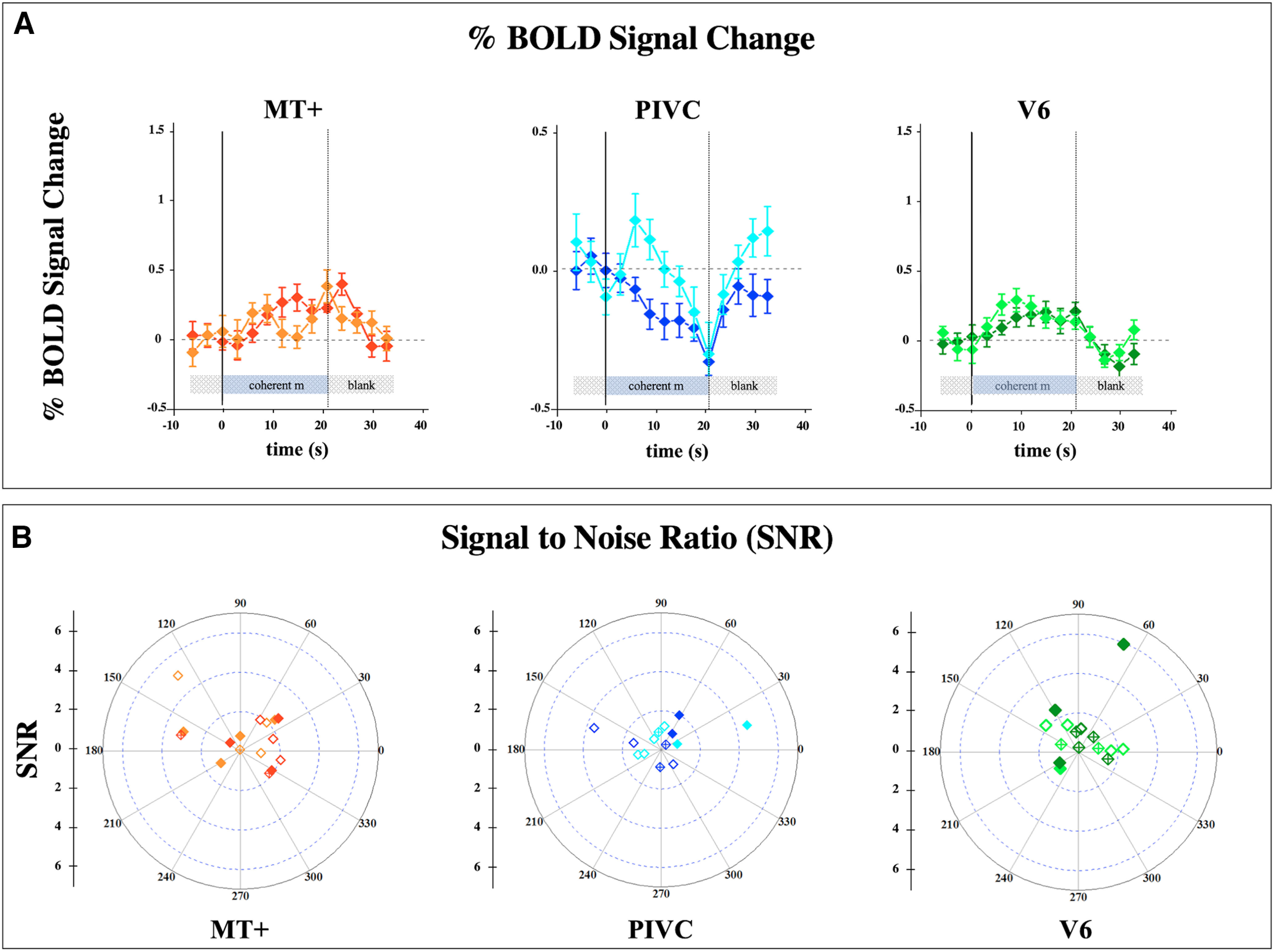
Responses of MT+, PIVC, and V6 areas to coherent flow motion versus blank. ***A***, Percentage BOLD signal change averaged across infants at 5 weeks of age. Other details are as in Figure 2. ***B***, The 2D scatter plots show the SNR and phases of the response estimated from the frequency analysis for each participant in the three areas. Open and solid symbols in the 2D plot indicate responses that are significant at *p* < 0.05 or *p* < 0.01, respectively. Cross symbols indicate nonsignificant responses. The dark and light color traces show the BOLD modulation of the left and right hemispheres, respectively.

Given the strong response to coherent versus random motion of MT+, PIVC, and V6 areas, we also measured the response of the same ROIs to coherent motion versus blank to evaluate whether the overall responsiveness of MT+, PIVC, and V6 areas to visual stimulation is particularly strong. Figure 5 shows the responses of MT+, PIVC, and V6 for the 5-week-old infants. The response to coherent motion versus blank is positive for MT+ and V6 and negative for PIVC, with BOLD increments and delays similar across areas. The amplitudes of the average response, pooled from left and right hemisphere, are equal to 0.17 ± 0.10%, −0.11 ± 0.11%, and 0.11 ± 0.12% for MT+, PIVC, and V6, respectively. A similar pattern of results was obtained in the 8 week infants with average amplitude of 0.16 ± 0.14%, −0.02 ± 0.08%, and 0.02 ± 0.06% for MT+, PIVC, and V6, respectively. The results that response amplitude is greater when contrasting coherent versus random motion than when contrasting coherent motion versus blank suggest that the random motion probably elicits a negative response in these areas. However, some caution should be taken in interpreting these data, given that the infant attention was not equally allocated during blank and motion, and sometimes during the blank period the infants drifted toward a presleep behavior.

**Figure 6.**
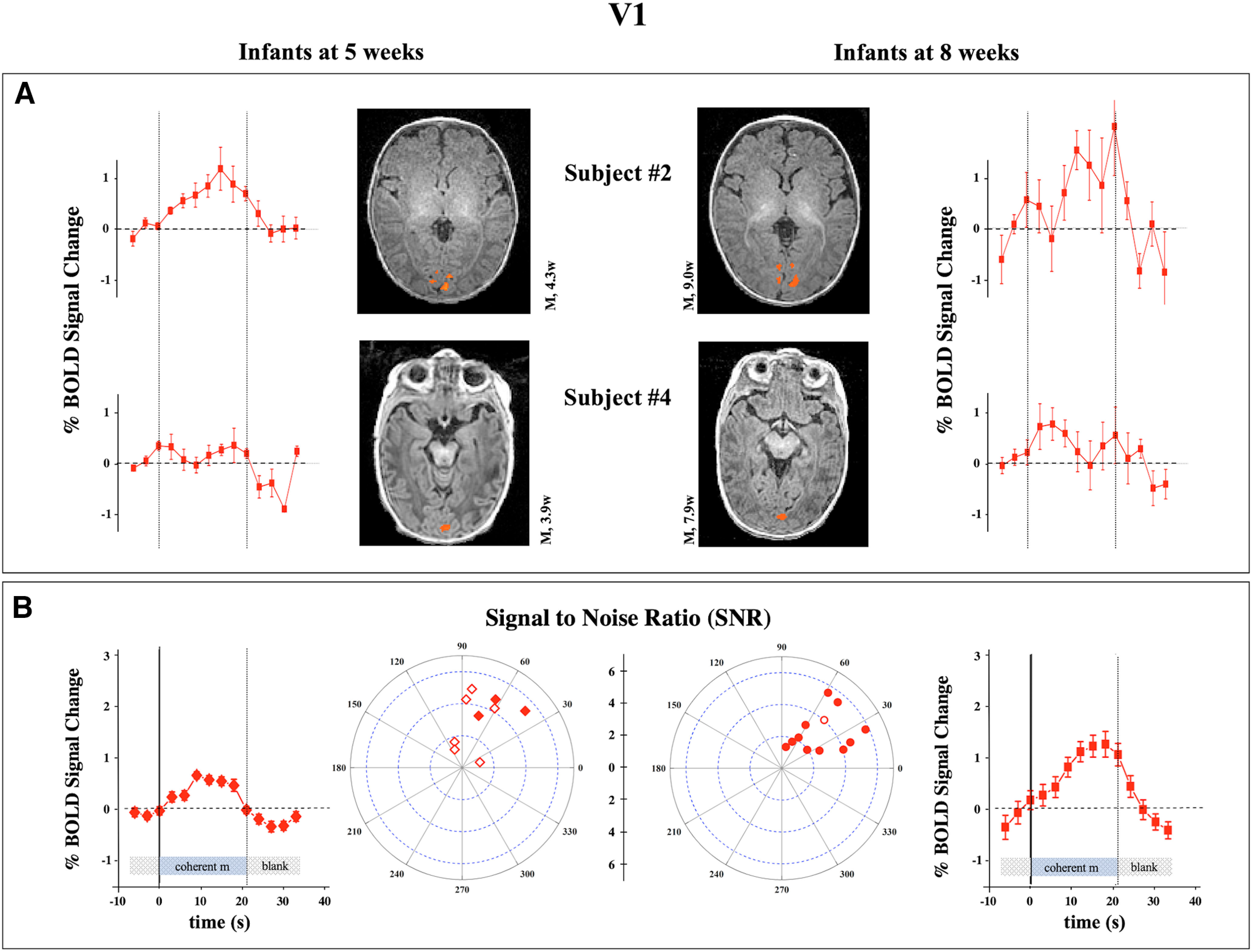
Responses of V1 to coherent versus blank. ***A***, ***B***, The analysis on two longitudinal recording (***A***) and of the full subject population (***B***) in response to moving stimuli versus blank for the primary visual cortex (V1) located along the calcarine sulcus. This area is subject to a maturation between the two ages with an increase of BOLD modulation of the ROI responses.

Occipital cortex, particularly voxels positioned along the calcarine sulcus, did not show a selective response to coherent flow with respect to random motion at 5 and at 8 weeks of age, whereas the response to visual stimuli against a blank screen was present and reliable in all subjects (Table 3). Figure 6*A* shows the responses along the calcarine sulcus (used to define the original mask; see above, Materials and Methods) in the two longitudinal subjects. The responses were smaller in amplitude and noisier in the younger age both for the three subjects measured longitudinally and for the group (average BOLD amplitude modulation, twice that of the 8-week-old infants, *p* = 0.008). Also, the extent of V1 was about half in the younger group, but given the great variance between subjects, the difference is not significant (*p* = 0.15).

We also attempted to perform an average group analysis to compare 5- versus 8-week-old infants, aligning all infant brains to a common anatomic atlas using the DARTEL approach. However, this was not possible given the quality of the T1 anatomic scanner for some subjects, which did not allow a good segmentation of the gray/white matter border with standard segmentation software in the younger infants.

### Functional connectivity

**Figure 7.**
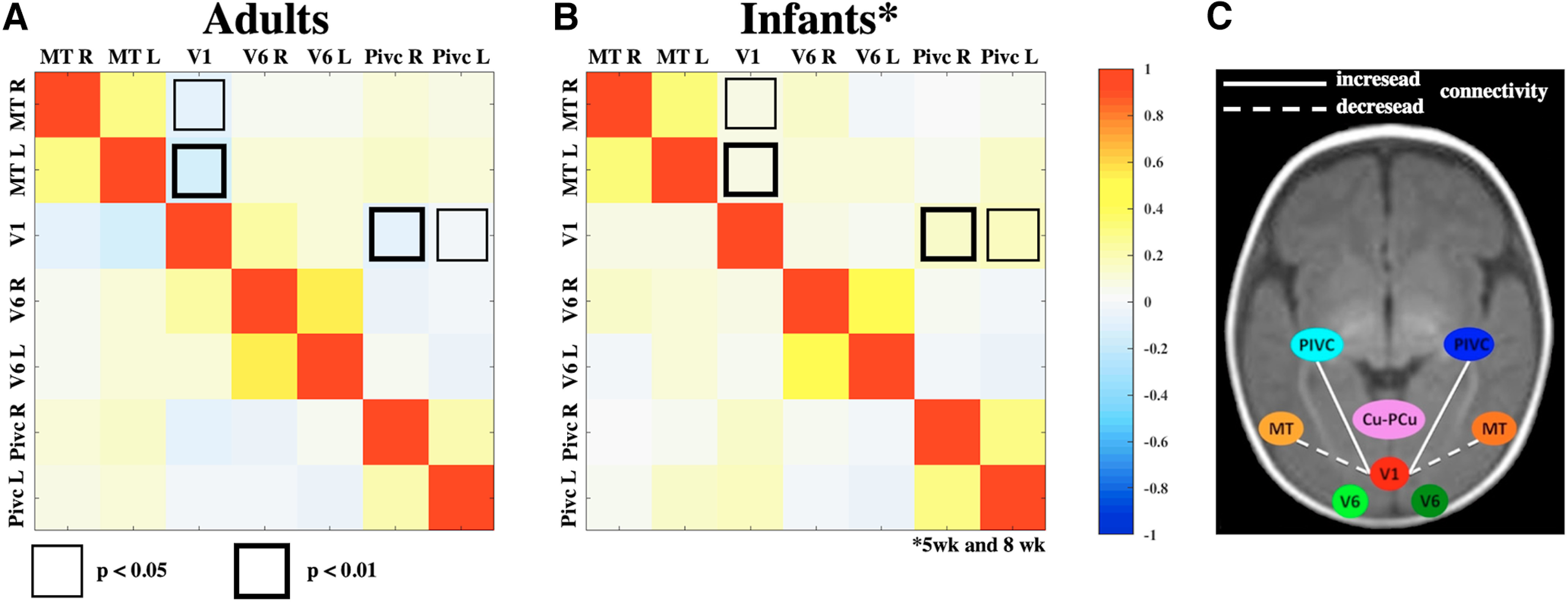
Comparison of functional connectivity for adults and infants. ***A***, ***B***, Correlation matrices of the BOLD resting-state activities among all possible ROIs in infants (***B***) and adults (***A***). Each value (scale at right) shows the average of the correlation across subjects. To test for differences between infant and adult correlation matrices, a two-tailed *t* test was performed between the two groups, and the checkboxes differently outlined above the main diagonal indicate the two significantly (at *p* < 0.01 and *p* < 0.05) different values. ***C***, The diagram illustrates the only two correlations that are different between adult and infants. R, Right; L, left.

Seven of the 5-week-old infants fell asleep in the scanner, and we were able to record resting-state BOLD activity. Given the sleep state, all infants had negligible head motion (Table 2), allowing a functional correlation analysis with the same preprocessing used for the visual response analysis. We could not apply more advanced techniques for artifact or noise reduction based on independent component analysis or generally of nuisance signal removal because these techniques required a good brain segmentation (not possible given the quality of our anatomic scans) or projection to a brain atlas, which is not available for perinatal ages. To gain information about the functional connection between motion-selective areas, we used the same ROIs defined on the response to visual stimuli, and we computed functional connectivity in the 5 week and in the 8 week infants. Given that the pattern of the results for each ROIs couple was not statistically different between the two age groups (*p* > 0.16) we combined the two datasets. The overall functional connectivity in infants (*n* = 13) is illustrated in Figure 7*B*, whereas the functional connectivity obtained in adults using similarly defined ROI is illustrated in Figure 7*A* (reproduced from Biagi et al., 2015). In infants a very strong functional connectivity across hemispheres is present in areas MT+, V6, and PIVC. However, the interhemispheric connectivity matrix is not statistically different from that in adults, suggesting an advanced development of callosal projections. In adults, MT+ and PIVC show a weak negative correlation with V1 activity (Fox et al., 2009) that is statistically different from that measured in infants. In infants these correlations are weak but positive. These results contrast with the adult-like value of functional connectivity in infants observed between V1 and V6.

## Discussion

The main result reported here is the absence of strong maturation of areas MT+ and PIVC between 5- and 8-week-old infants. This result contrasts with an increase in responsiveness in V1 and in V6 and the difficulty of locating other associative dorsal areas, like those along the POS, which respond well in 8-week-old infants but not in younger infants.

The lack of increase in BOLD response between 5- and 8-week-old infants in MT+ and PIVC is interesting. Both the response and the extent of these areas change very little with age, despite both areas being located in a lobe whose gray matter increases substantially in the first year of life (Gilmore et al., 2012). Motion perception is extremely rudimental at 5 weeks, whereas it is quite robust in 8-week-old infants (Wattam-Bell, 1992, 1996a,b; Braddick et al., 2005; Braddick and Atkinson, 2009; Armstrong et al., 2011; Maurer and Lewis, 2017), requires a precise timing of visual activity, and is disrupted in pathologic conditions where the transmission of visual responses from retina or thalamus is impaired (Guzzetta et al., 2009; Atkinson, 2017; Maurer and Lewis, 2017; Merabet et al., 2017; Bhat et al., 2021; Perani et al., 2021). Nevertheless, when using appropriate low spatial frequency and low velocity stimuli, newborns can react to expanding or contracting flow motion (Ball and Tronick, 1971; Náñez and Yonas, 1994), consistent with the similar BOLD response observed here between the two age groups. In 5-week-old infants myelinization is far from complete, reaching maturation only by ∼3 months of age (Dubois et al., 2008; Natu et al., 2021). Similarly, latency of the central visual evoked potentials (VEP) or ERP visual response changes dramatically around the first month of life (Fiorentini and Trimarchi, 1992; Morrone et al., 1996; Lee et al., 2013) and correlates with myelination (Dubois et al., 2008). The slow acuity development, and with the fact that contrast sensitivity in young infants is more similar to adult scotopic than photopic sensitivity (Fiorentini et al., 1980), strongly suggests a delayed maturation of foveal pathways. Our stimuli are very salient and clearly visible to the infants and should excite equally the foveal and the peripheral pathways. Although the motion response of V1 is prevalent for peripheral stimuli, the MT+ response is balanced between center and peripheral eccentricities. This may suggest that the early maturation of MT+ and PIVC reflects the prevalent input from peripheral visual field and/or from magnocellular input.

V6 shows important developmental changes between 5 and 8 weeks of age. At 8 weeks V6 shares a similar maturation to other associative visual areas, like V3 and V4 (Biagi et al., 2015; Kosakowski et al., 2022). All these associative areas have extended representation of the foveal region, and a more gradual development of foveal vision may generate the observed maturation change between these ages. Consistently, BOLD responses to coherent motion versus blank in primary visual cortex are subject to most dramatic changes between 5 and 8 weeks of age. This result is consistent with the recent evidence of retinotopic mapping of V1 in infants from 5 months, showing a reverse gradient of BOLD maturation from primary to associative areas (Ellis et al., 2021). Ellis et al. (2021) also addressed the question of whether the center visual field development followed a different maturation trajectory than peripheral visual field, reaching unfortunately inconclusive results.

The optimality of stimuli used to elicit the BOLD response plays a crucial role in establishing the developmental timeline of the various visual areas. Here, we optimized the velocity and size of the motion stimuli to elicit a strong response in our young subjects. The processing of stimuli with such large dots (1.3°) and slow velocity (5°/s) would be resilient to timing errors along the immature visual pathways. Use of stimuli close to the detection threshold may have produce a delayed maturation timeline, with responses in MT emerging later than in V1 (Kiorpes et al., 2012; Van Grootel et al., 2017). BOLD responses to highly salient stimuli with respect to blank in infant monkeys showed an earlier maturation of primary visual cortex with respect to associative areas and a gradual maturation that was completed only very late in adolescence (48 months for monkeys). This gradual increase of the response is consistent with our data on V1, which in 8-week-old infants is still 10 times smaller than in adults. Interestingly the maturation gap of V1 with respect to associative cortex in monkey MT+ decreased when using more complex and more appropriate stimuli for MT+ selectivity (Kiorpes and Movshon, 2014; Kiorpes, 2016). Another important aspect that may induce an apparent late maturation of MT+ BOLD in monkeys with respect to V1 is related to the unavoidable protocol of using anesthetized and paralyzed infant monkeys. Associative areas are more sensitive to levels of alertness in primates and in general to the level and type of anesthetic used.

The differential maturation between V1 and MT+ is also consistent with results in patients with perinatal damage. Many developmental pathologies with lesions in visual pathways or with cataracts are associated with a specific deficit in motion perception (for review, see Maurer and Lewis, 2017; Atkinson, 2017), thought to reflect malfunctioning of MT+ or other dorsal associative cortices. However, this prevailing idea have been questioned recently by the results of studies that measured BOLD response to motion (Rezk et al., 2021), cortical thickness (Bhat et al., 2021), or white matter alteration (Perani et al., 2021) in a variety of neurodevelopmental disorders. These studies show that the perceptual motion deficit correlates with anatomic or BOLD response of V1 rather than MT+. This suggests that much of MT+ development may be independently affected by diseases that damage V1, probably because MT+ relies on different inputs at least during development. This is consistent with marmoset MT developmental data that receive, at perinatal age, a strong direct input from the retina that bypasses V1 (Warner et al., 2012; Bourne and Morrone, 2017).

The previous discussion points to a differential BOLD response maturation of MT+ and PIVC that can be explained in terms of foveal peripheral and/or parvo/magno-pathway differential development. Similar explanations can also be used to interpret the functional connectivity results. No major differences were observed between 5 and 8 weeks of age in the connectivity maps. This is consistent with previous reports suggesting that average functional connectivity changes slowly in the first years of age (Gao et al., 2015; for review, see Gilmore et al., 2018). However occipital cortex and, in particular, V1 undergoes a more rapid functional connectivity change in the first 6 months postnatal. Interestingly, in the second month of life the connectivity between V1 and a region that in our infants includes MT+ is starting to emerge (Gilmore et al., 2018). In addition, in the first 6 months, an anticorrelation between V1 and V2 and the insular cortex develops (Fransson et al., 2011) consistent with the observed change demonstrated here between V1 and PIVC (Fig. 5). Overall, the immature connectivity between V1 with MT+ and PIVC can be interpreted as a different (magno/parvo or fovea/peripheral) input innervating these structures in the first 2 months of life and in adults.

Although the maturation of connectivity observed in our data are highly consistent with the data reported in the literature, acquiring infant resting-state data during wakefulness is extremely difficult. We tried several times, but even small movements, like pacifier sucking, induce strong BOLD correlations, making the method unreliable. We recorded rs-fMRI during sleep but could not classify the sleep phase. MRI functional connectivity during wakefulness and sleep is very different, with basic properties like thalamic visual cortical lag being altered during sleep (Mitra et al., 2017). It is possible that the sleep state may affect more profoundly V1, and the developmental difference in connectivity that we detected may be a consequence of the sleep effect. A near infrared spectroscopy (NIRS) study has shown a general higher functional connectivity during sleep than during wakefulness in infants (Taga et al., 2018), and this may mask the anticorrelation connection with MT+.

The differential development between areas could also reflect an immaturity in the neurovascular system that may not be homogeneous across cortices. NIRS and BOLD data consistently report positive BOLD responses during stimulation. Our data show a slower hemodynamic response at 5 weeks that does not vary across areas. This suggests a similarity of the neurovascular properties of these areas at 5 weeks. However, the issue is very complex (Kozberg and Hillman, 2016), and the maturation of the neurovascular properties may contribute to explain some of the present and published connectivity results.

Cortical thickness changes gradually during development. It is around 1.5 mm in occipital area, reaching ∼2.3 mm by the first year (Li et al., 2015). However, nearly all occipital poles, including the mediotemporal portion, have about the same thickness at birth, which is unlikely to explain the different maturation timelines observed here. Given the large voxels used in comparison to the thickness, it is also improbable that BOLD data are altered by partial volume effects. At birth T1 signal (inversely related to myelin fraction) and mean diffusivity systematically increase from V1 to higher tiers along the dorsal stream. The rate of changes of myelination is higher in intraparietal areas of the dorsal stream between the first and the third month of age, suggesting a faster maturation of the intraparietal cortices with respect to primary visual cortex (Natu et al., 2021). Unfortunately, no data are available at the finer scale allowing us to compare it with the BOLD development observed here.

We present the first evidence that the visual cortical network for motion processing has already achieved a high specialization by 5 weeks of age. The high selectivity of MT+ and PIVC to motion contrasts with the poor behavioral performance in motion discrimination at this early age and the immaturity of functional connection of these cortical areas with V1. A possible interpretation of this apparent discrepancy may be the greater contribution of the motion area of the peripheral visual field, which may have a faster developmental trajectory. Overall, our data demonstrate the feasibility of studying visual cortical maturation in 4- to 5-week-old infants and show that development of cortical selectivity motion can be used as a benchmark to evaluate brain disorders.
